# Understanding ion-induced assembly of cellulose nanofibrillar gels through shear-free mixing and *in situ* scanning-SAXS†

**DOI:** 10.1039/d1na00236h

**Published:** 2021-07-19

**Authors:** Tomas Rosén, Ruifu Wang, HongRui He, Chengbo Zhan, Shirish Chodankar, Benjamin S. Hsiao

**Affiliations:** Department of Chemistry, Stony Brook University Stony Brook New York 11794-3400 USA trosen@kth.se benjamin.hsiao@stonybrook.edu; Department of Fiber and Polymer Technology, KTH Royal Institute of Technology SE-100 44 Stockholm Sweden; Wallenberg Wood Science Center, KTH Royal Institute of Technology SE-100 44 Stockholm Sweden; National Synchrotron Light Source II, Brookhaven National Lab Upton NY USA

## Abstract

During the past decade, cellulose nanofibrils (CNFs) have shown tremendous potential as a building block to fabricate new advanced materials that are both biocompatible and biodegradable. The excellent mechanical properties of the individual CNF can be transferred to macroscale fibers through careful control in hydrodynamic alignment and assembly processes. The optimization of such processes relies on the understanding of nanofibril dynamics during the process, which in turn requires *in situ* characterization. Here, we use a shear-free mixing experiment combined with scanning small-angle X-ray scattering (scanning-SAXS) to provide time-resolved nanoscale kinetics during the *in situ* assembly of dispersed cellulose nanofibrils (CNFs) upon mixing with a sodium chloride solution. The addition of monovalent ions led to the transition to a volume-spanning arrested (gel) state. The transition of CNFs is associated with segmental aggregation of the particles, leading to a connected network and reduced Brownian motion, whereby an aligned structure can be preserved. Furthermore, we find that the extensional flow seems to enhance the formation of these segmental aggregates, which in turn provides a comprehensible explanation for the superior material properties obtained in shear-free processes used for spinning filaments from CNFs. This observation clearly highlights the need for different assembly strategies depending on morphology and interactions of the dispersed nanoparticles, where this work can be used as a guide for improved nanomaterial processes.

## Abstract 2

Using *in situ* X-ray scattering in a shear-free mixing experiment, the time-resolved ion-induced gel transition of cellulose nanofibrils is studied.

## Graphical abstract

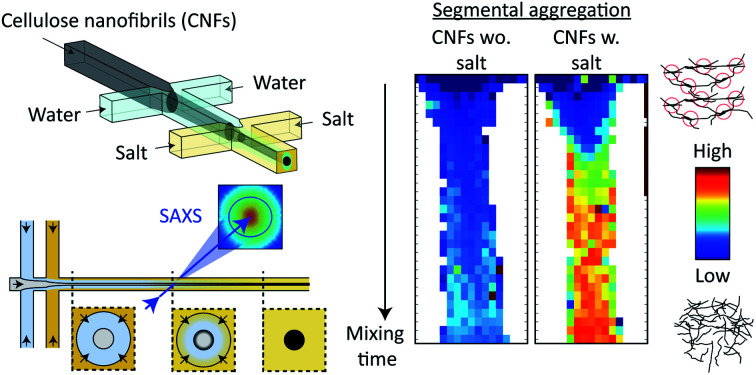

## 1 Introduction

With the grand challenge of dealing with global warming and unsustainable usage of synthetic polymers, there is a growing demand for advanced materials extracted from natural resources that are both biobased and biodegradable, which could provide the backbone for a circular bioeconomy. Using nanoparticles from cellulose (nanocellulose), the most abundant natural polymer on earth, as building blocks for new material development, this approach has the potential to meet this demand owing to impressive mechanical properties, biocompatibility and simplicity for chemical modification and functionalization of these materials.^1–3^ Nanocellulose can be extracted from any lignocellulosic feedstocks, including wood, non-woody plants (agricultural residues, shrubs and weeds) and algea, some are considered zero or even negative values to the communities.^4,5^

*Nanocellulose* extracted from lignocellulose biomass using the top-down defibrillation process can be divided into two types: cellulose nanocrystals (CNCs) and cellulose nanofibrils (CNFs).^1^ CNCs are most often extracted through acid hydrolysis, resulting in negatively charged nanorods that are short (100–300 nm), flat (widths 10–30 nm, thickness 3–5 nm) and rather monodisperse.^8^ CNFs can be obtained through cellulose oxidation and high-pressure homogenization, also resulting in negatively charged nanofibrils that are long (500–2000 nm), thinner (widths 2–10 nm, thickness about 2 nm), highly polydisperse, and often with pronounced branching and kinks.^9–13^ The differences in morphology leads to very different behavior in dispersions, thus making CNFs and CNCs not suitable in the same applications. Owing to their high crystallinity and chiral nematic organization at high concentrations, CNCs are particularly interesting for tuning optical properties of materials,^14,15^*e.g.* 2D films in photonic applications.^16^ The main advantage of CNFs, are their inherent ability to form volume-spanning networks at very low concentrations,^17–22^ making them highly desirable as building blocks in membranes, barriers, aerogels, foams,^1,2,4^ 3D-printed materials^23^ and also for making filaments that can be used for reinforcing new biobased composites and high-performance textiles.^22,24–32^

The properties of materials produced from the bottom-up re-assembly of CNCs and CNFs are highly dependent on their spatial and orientation distributions within the material, which in turn are set by the processing conditions.^14,29^ A common route for controlling the nanostructure of the final product includes hydrodynamic alignment with subsequent assembly (transition from dispersion to gel, *i.e.* gelation) by neutralizing the electrostatic repulsion between nanoparticles through the addition of salts or acids.^25,28,29^ It has been found during spinning filaments from CNFs, that a system, which is aligned through a pure extensional flow with no shear, is highly desirable to obtain optimal mechanical properties.^28,29^ Nanoscale structures and assembly of CNFs and CNCs are commonly studied by small-angle X-ray scattering (SAXS) of pre-mixed samples of CNFs^6,9,33,34^ or CNCs.^35–37^ However, in order to understand fully why a certain process can lead to desirable material properties, *in situ* characterization on the nanometer scale is essential.

Although the nanostructure during hydrodynamic alignment (*without* assembly) of CNFs and CNCs has been studied before using *in situ* SAXS,^26,28,38,39^ there are no previous *in situ* SAXS studies that focus on characterization of time-resolved nanoscale kinetics during combined alignment and assembly of CNFs. Such knowledge is central in understanding how to fabricate nanostructured materials from nanocellulose on a larger scale. Håkansson^26^ combined *in situ* SAXS (*without* assembly) with polarized optical microscopy (POM) (*with* assembly) to study the CNFs during the structural transition. Even though the qualitative effect of average alignment could be determined with POM, the *in situ* SAXS experiment needed to be performed without a gel-transition due to “*clogging in the channel and limited optical access*”.

In our recent work,^7^ we demonstrated a shear-free mixing experiment combined with the scanning-SAXS technique to resolve nanoscale kinetics of CNCs during hydrodynamic alignment and ion-induced assembly. As CNCs in a semi-dilute state form locally arranged regions, called *tactoids*,^40,41^ their collective behavior aids in overcoming Brownian rotary diffusion and thus aligns very well at low flow rates.^7,39^ When a sodium chloride solution is added to the system, the positive Na^+^ ions screen the surface charges, and CNCs can assemble into a connected network and form a gel state. However, since Na^+^ ions also disturb the tactoidal arrangement, rotary diffusion quickly makes the material isotropic prior to the gel formation, highlighting the importance of controlling the time scales in the CNC process.

**Fig. 1:**
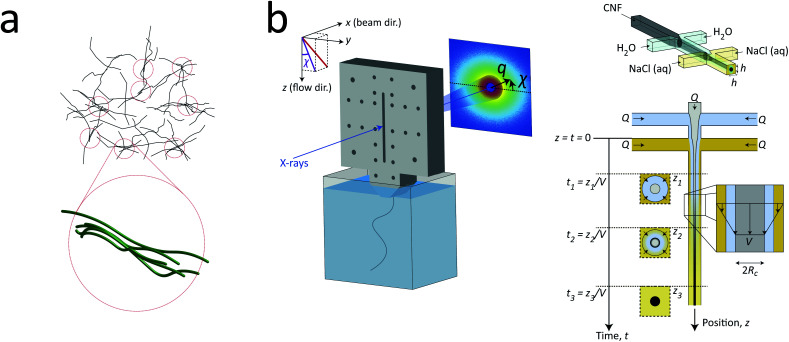
Description of the experiment in the present work. (a) Gels of cellulose nanofibrils (CNFs) provide an irregular network structure with locally aligned and entangled regions called *segmental aggregates*.^6^ (b) Schematic illustration of the shear-free flow-focusing mixing setup in the present work. Dispersed CNFs are focused by buffer sheath flows of water, whereby the core dispersion is detached from the walls and additional sheath flows with salt solution initiates the transition to a gel. All the inlet channels have the same flow rate *Q* = 0.5 mL h^−1^ and all channels have square cross-section with side *h* = 1 mm. The detached flow has constant velocity *V* during the transition, where mixing times can thus be extracted from downstream positions *z* through *t* = *z*/*V*. The temporospatial nanoscale kinetics during the transition is obtained through SAXS by focusing the beam at various positions in the flow. The flow cell is placed with *z* in the same direction as gravity allowing the gel to freely eject into a container with water. The coordinate system is further defined using *x* as the beam direction and *y* as the cross-streamwise direction. The projected orientation of a fiber w.r.t. the *z*-axis in the viewing (*yz*) plane is given by *χ*, which is represented on the detector as increased scattering at an angle *χ* from *y*-direction. Partly reproduced from earlier work^7^ with permission from the Royal Society of Chemistry.

In the present work, we applied exactly the same method and setup as described in our previous work^7^ to study the nanostructure of CNFs during hydrodynamic alignment and assembly into a gel, *i.e.* revisiting the original idea by Håkansson.^26^ We found that, while CNC assembly is dominated by enhanced rotary diffusion during assembly, the CNF assembly is dominated by mechanical entanglement and the formation of segmental aggregates, *i.e.* locally aligned and entangled segments of CNFs providing a network structure that suppresses rotary diffusion (see Fig. 1a).

## 2 Results and discussion

To determine the time-resolved nanostructural changes during the ion-induced sol–gel transition of CNF, we apply the same shear-free mixing experiment combined with the scanning-SAXS technique as demonstrated in earlier work,^7^ which is illustrated in Fig. 1b. Here, a flow of dispersed CNFs is firstly focused by two perpendicular flows of water. Owing to the viscosity ratio and effective interfacial tension between water stream and CNF dispersion stream, the core flow is detached from the walls and completely surrounded by a water;^42^ a layer that also acts as a buffer between the gelation agent (*e.g.* ions) and core dispersion. Two additional sheath flows with a salt solution are also added, which further causes additional focusing of the flow and initiates gelation as ions diffuse through the buffer layer of water into the core CNF dispersion. Since the core is completely detached from the walls, the CNF dispersion is not affected by any velocity gradients and flowing with constant velocity *V*. Such conditions allow the downstream locations *z* to be directly converted to mixing times through *t* = *z*/*V*.

The flow rates in the setup were determined using the same considerations as done before^7^ with respect to maximizing scattering contrast and ensuring core detachment, where a good compromise was found by applying the same flow rate *Q* in all five inlet channels. Ideally, the setup is running at as low flow rates as possible to maximize the probed mixing times within the visible portion of the channel and ensure that a gel is formed within this region. However, the inertia in the flow has a stabilizing effect, where a too low flow rate is therefore practically not possible. For the CNF dispersion, the lowest practical flow rate was *Q* = 0.5 mL h^−1^ and was used in this study. Through complementary experiments using POM, the core flow was found to be circular with radius *R*_c_ = 0.185 mm and velocity *V* = 1.3 mm s^−1^ at the given experimental conditions, which provided the maximum mixing times of 20 s inside the visible portion of the mixing flow cell (see Method section and ESI† for details). With the same POM-experiments, we could also determine that a gel was formed as it exited the flow cell. In contrast to dispersed CNF, the gel–water interface will be visible in the outlet container and will not diffuse out to the surrounding over time (see ESI Movie†).

With this stationary shear-free mixing experiment, the time-resolved kinetics of the structural transition was obtained by scanning-SAXS, where a focused X-ray beam was aimed at various portions of the channel and the scattered X-ray intensity *I* was analyzed as a function of scattering vector *q*, corresponding to spatial frequencies of electron density differences in the beam, and azimuthal angle *χ*. The scattering profile *I*(*q*, *χ*) thus could reveal any structural features in the range roughly between 1–100 nm (see Method section for details).

**Fig. 2:**
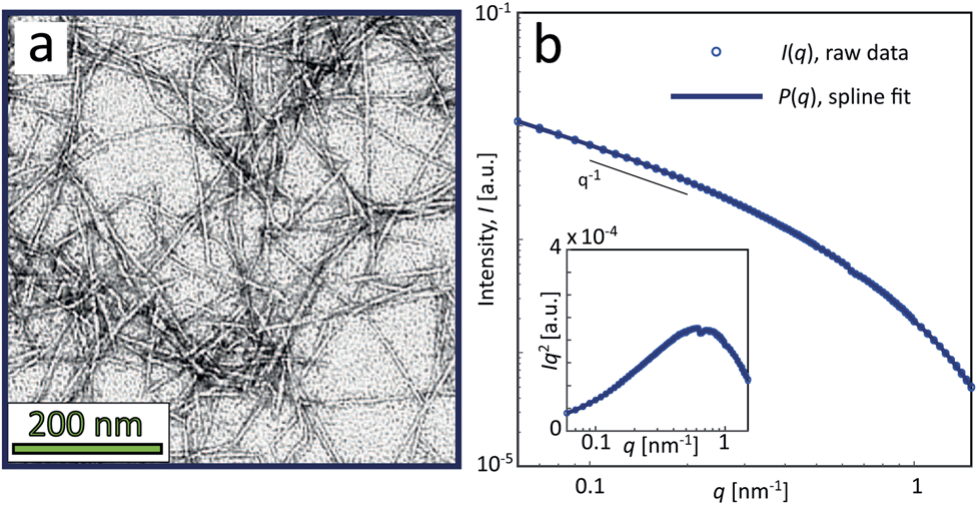
Characterization of CNF dispersions used in the present study (identical dispersions were used in earlier work^39^). (a) TEM of CNFs (reprinted with permission); (b) isotropic SAXS intensity profiles *I*(*q*) of CNF dispersion at 0.4 wt%. The circles show the combined raw data from five different measurements and the thick curve is a spline fit to the data used as a form factor *P*(*q*) in this study. The inset shows the Kratky plot of the same data.

The sample used in this work was a commercially available sample, consisting of TEMPO-oxidized charge-stabilized CNFs dispersed in water, from the Process Development Center of University of Maine. This sample was identically prepared as in earlier work^39^ at a concentration of 0.4 wt%. This sample was also comparable to what has been used in fiber spinning processes of CNFs.^29^ A TEM image of these nanofibrils is shown in Fig. 2a, where the lengths range from 200–1000 nm. The scattering intensity obtained from SAXS of CNFs in an isotropic dispersed state is illustrated in Fig. 2b. As the lengths of the CNFs do not contribute to the scattering in the experimental *q*-range and the concentration is low (no structure factor), the isotropic scattering profile *I*(*q*) is reflecting the mean cross-sectional shape and size of the individual CNF, which will be used as the *form factor P*(*q*) in this work. Using this form factor of CNFs, the average cross-sectional dimensions were determined in earlier work^39^ to be 5.6 × 2.0 nm^2^. Any aggregation of CNFs where fibril segments are in close proximity,^6^ will show up as excess scattering with *I*(*q*) > *P*(*q*) at low values of *q*. This is likely to occur with the addition of an aqueous NaCl solution, where the added Na^+^ ions will screen the negative charges of CNFs and the fibrils are attracted to each other by van der Waals forces; a process that leads to the formation of a connected fibrillar network and a transition to a gel. In this work, this transition is initiated by using a 200 mM NaCl solution as the gelation agent in the mixing experiment (see Fig. 1).

**Fig. 3:**
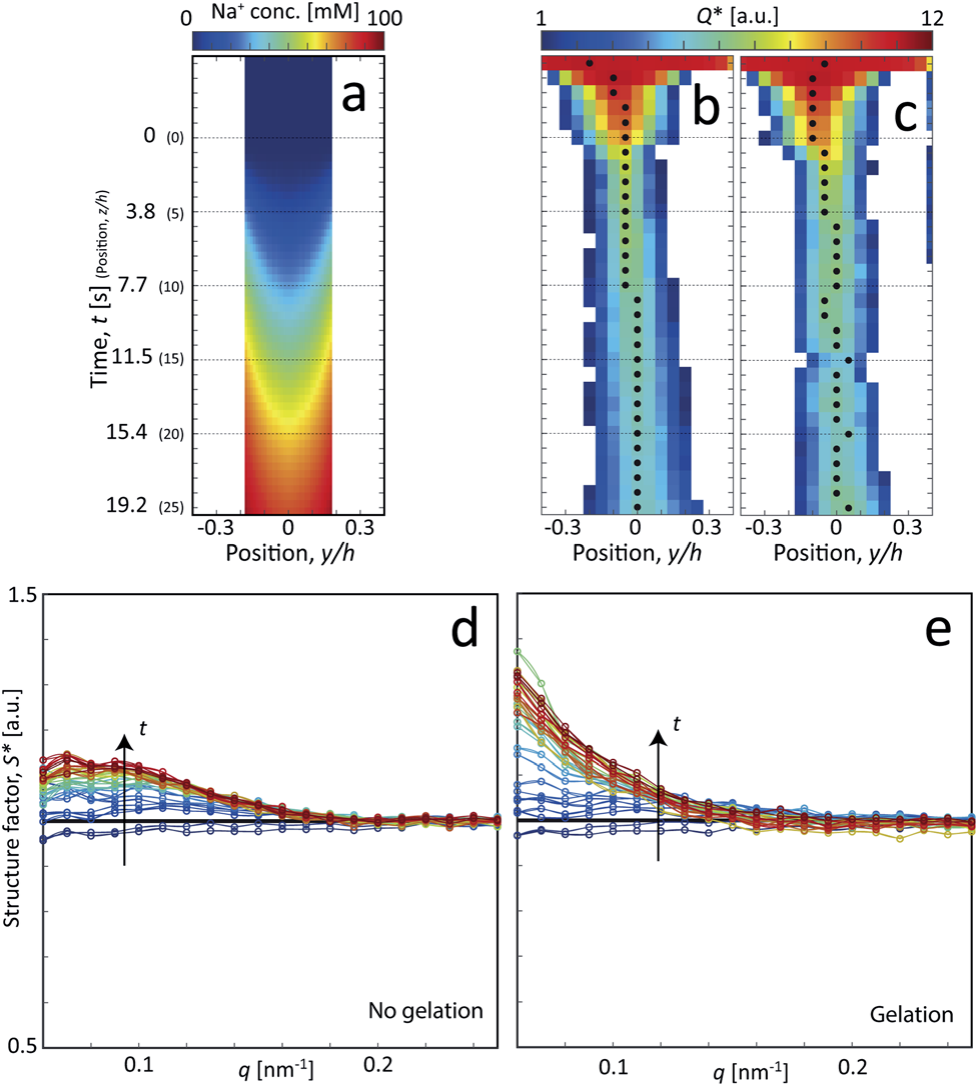
Results of the scanning-SAXS of CNFs. (a) Local Na^+^ concentration in the core from the ion diffusion simulation averaged in the beam (*x*) direction. (b and c) Invariant Q* depending on position *z* and *y*, where the downstream position *z* is converted to time using *t* = *z*/*V* for flow: (b) with no induced gelation, and (c) with gelation. The markers indicate the positions of maximum Q* used as the centerplane data. (d and e) Structure factor S* along the centerplane, *i.e.* depending on time *t* (indicated by arrows) for flow with for flow (d) with no induced gelation, and (e) with gelation.

Following the same procedure as done previously,^7^ the Na^+^ concentration in the channel (averaged in the beam direction) was estimated in the flow-focusing mixing experiment using the experimentally determined values of *V* and *R*_*c*_. This is illustrated in Fig. 3a, where we find that the Na^+^ concentration will almost reach 100 mM at the most downstream location in the channel (see Method section for details).

The *in situ* scanning SAXS experiments were performed at the LiX beamline (16-ID) at the National Synchrotron Light Source II (NSLS-II) in Brookhaven National Laboratory (BNL), USA. The mixing flow cell was mounted on a traversing stage, allowing the beam to be focused at different locations in the channel. Initially, the invariant Q* was calculated for every SAXS image, reflecting the amount of CNFs in the beam. This quantity could thus provide an image of the flow as seen in Fig. 3b without gelation (water in the second focusing) and in Fig. 3c with gelation (salt solution in the second focusing). The centerplane locations were determined at each downstream location *z* as the positions with highest invariant and noted with the black symbols in Fig. 3b and c. For the centerplane data, the structure factor S* = I*/P* was determined, where the asterisk denotes a normalization of the data with its invariant, i.e. I* = *I*/Q*. The structure factor S* is a measure of the deviation from the form factor (where S* = 1) and can be used to analyze the ion-induced structures in the system. The results without gelation and with gelation are illustrated in Fig. 3d and e, respectively.

**Fig. 4:**
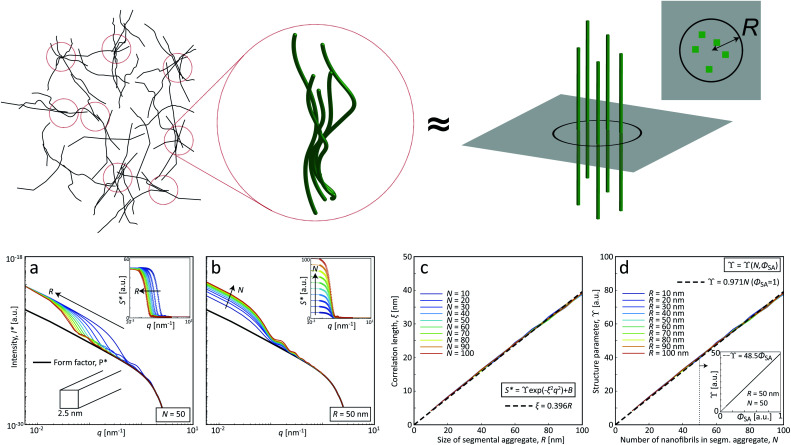
SAXS-simulations of segmental aggregates, where locally aligned and entangled CNFs can be modeled by just considering the scattering from an average cross-sectional distribution of *N* square CNFs (side 2.5 nm) randomly placed inside a circular aggregate of radius *R* and averaging over 100 configurations for each parameter pair of *N* and *R*. (a and b) Simulated SAXS-curves I*(*q*) (normalized by I* = *I*/*N*) and corresponding structure factor S* (insets) with (a) varying *R* at *N* = 50, and (b) varying *N* at *R* = 50 nm. The solid curves in insets show the fit results assuming S* = *ϒ* exp(−*ξ*^2^*q*^2^) + *B*. (c) Correlation length *ξ*(*R*) found by fitting S* for various *N* with dashed line is a linear fit of *ξ* ≈ 0.4*R*. (d) Structure parameter *ϒ*(*N*) at a volume fraction of segmental aggregates *Φ*_SA_ = 1 from the same fit for various *R*; inset showing a linear relationship between *ϒ* and *Φ*_SA_ demonstrating that *ϒ* ≈ *NΦ*_SA_.

Although quite subtle, there are some changes in the structure factor worth noting in both cases, which both show excess scattering at low *q*. Geng *et al.*^6^ attributed the excess scattering at low *q* to the formation of segmental aggregates within the CNF gel when adding NaCl, *i.e.* locations within the gel where few nanofibrils are locally connected in denser regions to form a network due to van der Waals attraction, illustrated in Fig. 4. In order to quantify the segmental aggregates, we developed a model using simplified SAXS-simulations where each segmental aggregate can be assumed to consist of *N* = 10, 20…100 parallel nanofibril elements (modelled with a 2.5 × 2.5 nm^2^ cross-section) that are randomly sampled within a circle of radius *R* = 10, 20…100 nm (details of the simulations are found in the Methods section). For each choice of *N* and *R*, the isotropic scattering intensity *I*(*q*) of a total of 100 different random configurations where averaged and plotted in Fig. 4a and b (the normalization in the simulations is done through I* = *I*/*N*). The structure factor S*(*q*) is extracted the same way as in the experiment, which is found to be very well fitted to a function S* = *ϒ* exp(−*ξ*^2^*q*^2^) + *B* (baseline *B* ≈ 1). The result is closely connected to the approach by Debye *et al.*^43^ applied by Geng *et al.*^6^ to study segmental aggregates in CNF gels. Here, we found quite intuitively that *R* only affects the correlation length *ξ* (see Fig. 4c) with a relationship *ξ* ≈ 0.4*R* for all choices of *N*. Similarly, we find that and that the structure parameter *ϒ* only depends on *N* (see Fig. 4d), where *ϒ* ≈ *N* for all choices of *N*. However, this is assuming that the entire dispersion consists of such aggregates, which is likely not the case. The effect of the volume fraction of CNF-material contained in segmental aggregates *Φ*_SA_ is shown in the inset in Fig. 4d, demonstrating a linear dependence up to the value of *ϒ*(*N*). The structure parameter can thus be written as *ϒ* ≈ *NΦ*_SA_, but we cannot determine if a high value of *ϒ* corresponds to high number of CNFs per segmental aggregate (high *N*) or high volume fraction of segmental aggregates (high *Φ*_SA_). In the remainder of this work, we extract the radius *R* and the structure parameter *ϒ* by fitting S*(*q*) = *ϒ* exp(−(0.4*R*)^2^*q*^2^) + *B*, and use these parameters to describe size of aggregates and the degree of aggregation, respectively.

**Fig. 5:**
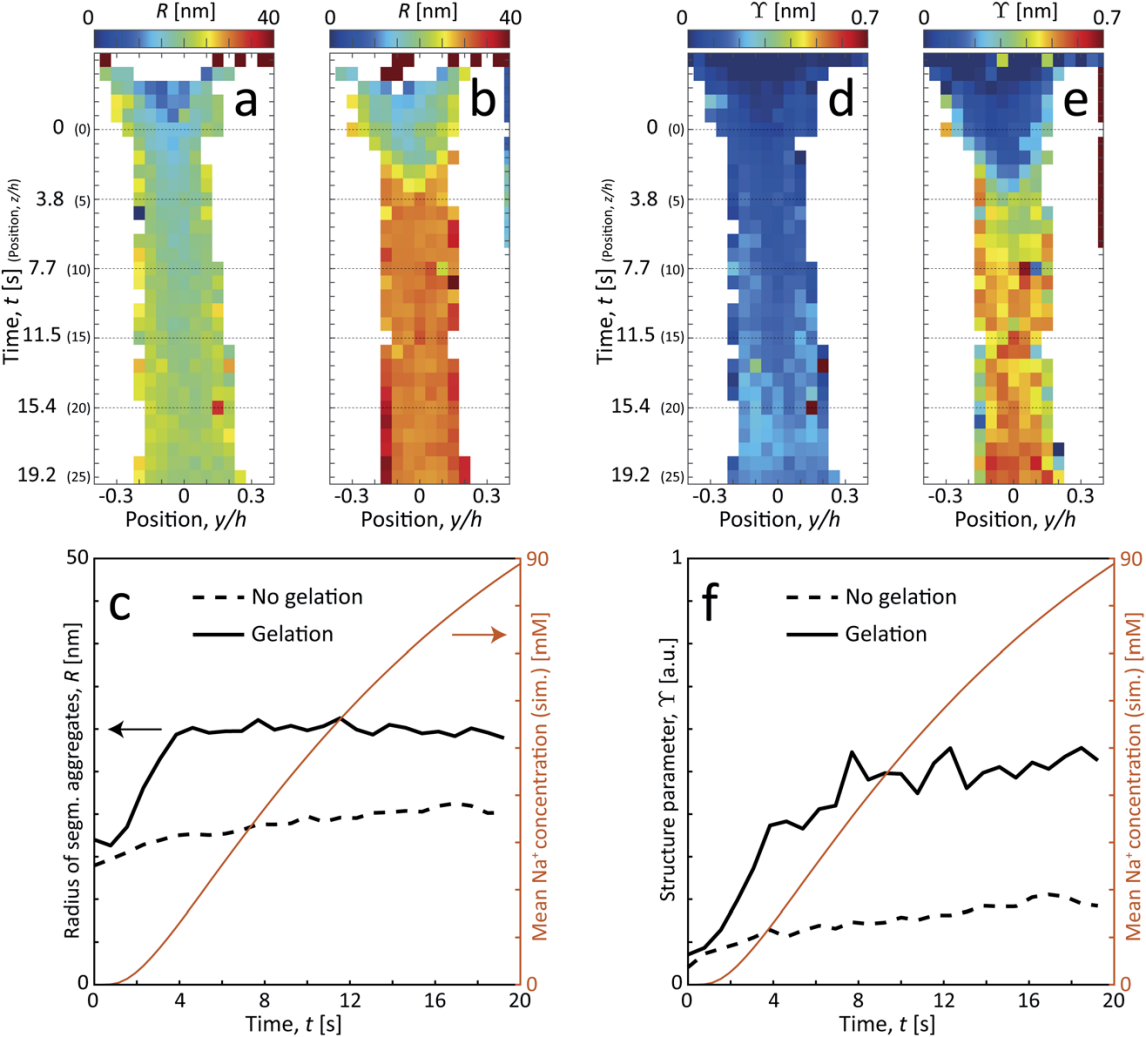
Results of scanning-SAXS mapping the formation of segmental aggregates in the CNF dispersion. (a and b) Radius of segmental aggregates *R* and (d and e) structure parameter *ϒ* for flow (a and d) with no induced gelation, and (b and e) with gelation. (c and f) The same parameters evaluated along the centerplane and compared with the simulated mean Na^+^ concentration 〈*c*〉.

The size of segmental aggregates *R* and degree of aggregation through *ϒ* were obtained from the scanning-SAXS experiment with and without gelation as illustrated in Fig. 5. Clearly, the addition of salt has a strong effect on the segmental aggregate formation, where the aggregate size quickly reaches a level of *R* ≈ 30 nm already after *t* = 4 s corresponding to an Na^+^ concentration of 〈*c*〉 ≈ 10 mM, but then remains constant even with increasing concentration. At this point the structure parameter has reached *ϒ* ≈ 0.4, where it also starts leveling off. The reason for the aggregates not increasing further in either size or amount, is probably due to the stiffness of the nanofibrils not allowing the network to deform in such a way so that new segmental aggregates can form. The simulated Na^+^ concentration at this point would correspond to around 10 mM, corresponding to a charge ratio of 1–2. This is consistent with the study of Håkansson *et al.*,^25^ who argued that a Na^+^ concentration of at least 10 mM was needed for a CNF gel to form.

Without the addition of salt (no gelation), the level of aggregation and aggregate sizes are very low. However, even without ion-induced gelation, it is seen that some segmental aggregates are formed from the extensional flow with a structure parameter reaching *ϒ* ≈ 0.2 with radius *R* ≈ 20 nm. This is likely caused by mechanical entanglements in a loose network as the stretched CNFs are subject to Brownian rotational motion.

**Fig. 6:**
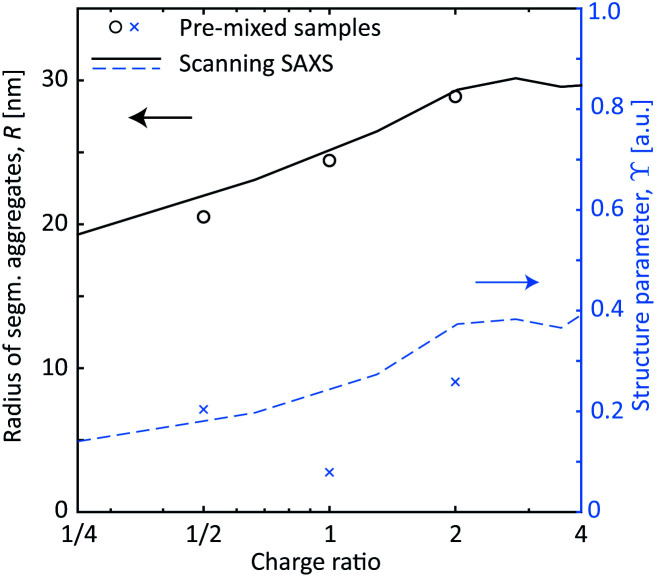
The SAXS results with pre-mixed samples of CNF gels (symbols) compared with the scanning-SAXS experiments (curves) using the simulated concentration 〈*c*〉. The black circles and solid curve show the radius *R* of segmental aggregates against the charge ratio of Na^+^ ions compared to the surface COO^−^ groups. The blue crosses and dashed curve show the structure parameter *ϒ* against charge ratio.

These *in situ* scanning-SAXS experiments revealed the kinetics of the CNF gel formation as the NaCl solution was added to the system. For comparison, gels were also prepared by pre-mixing the dispersion with different amounts of NaCl as described in Table 1 and leaving them for more than 12 hours prior to the SAXS measurements. The structure factor S* was obtained in the same way as described before, from which the resulting radii *R* of segmental aggregates and structure parameters *ϒ* were obtained. These quantities were then compared with the gel formed in the time-resolved scanning-SAXS experiment in Fig. 6, using the simulated mean Na^+^ concentration 〈*c*〉 to calculate the charge ratio. Interestingly, in the pre-mixed samples, *R* increased with the charge ratio in the same manner as in the experiment and reached the same value of *R* ≈ 30 nm. The structure parameter *ϒ* on the other hand did not seem to follow the same trend with respect to the charge ratio and the corresponding values were slightly lower than the ones found in the flowing system at a given charge ratio. It seems thus that the size of the segmental aggregates in the CNF dispersion is only dependent on the salt concentration, but the amount of such aggregates will depend on how the CNF network is deformed during gel formation. As was seen, the extensional flow itself induces segmental aggregation and the combination with higher salt concentration likely caused nanofibrils to aggregate more often.

**Table 1:** List of pre-mixed samples to compare with the *in situ* scanning-SAXS experiment

#	Surface charge, [mM]	Na^+^ added, [mM]	Charge ratio
1	6	3	0.5
2	6	6	1
3	6	12	2

**Fig. 7:**
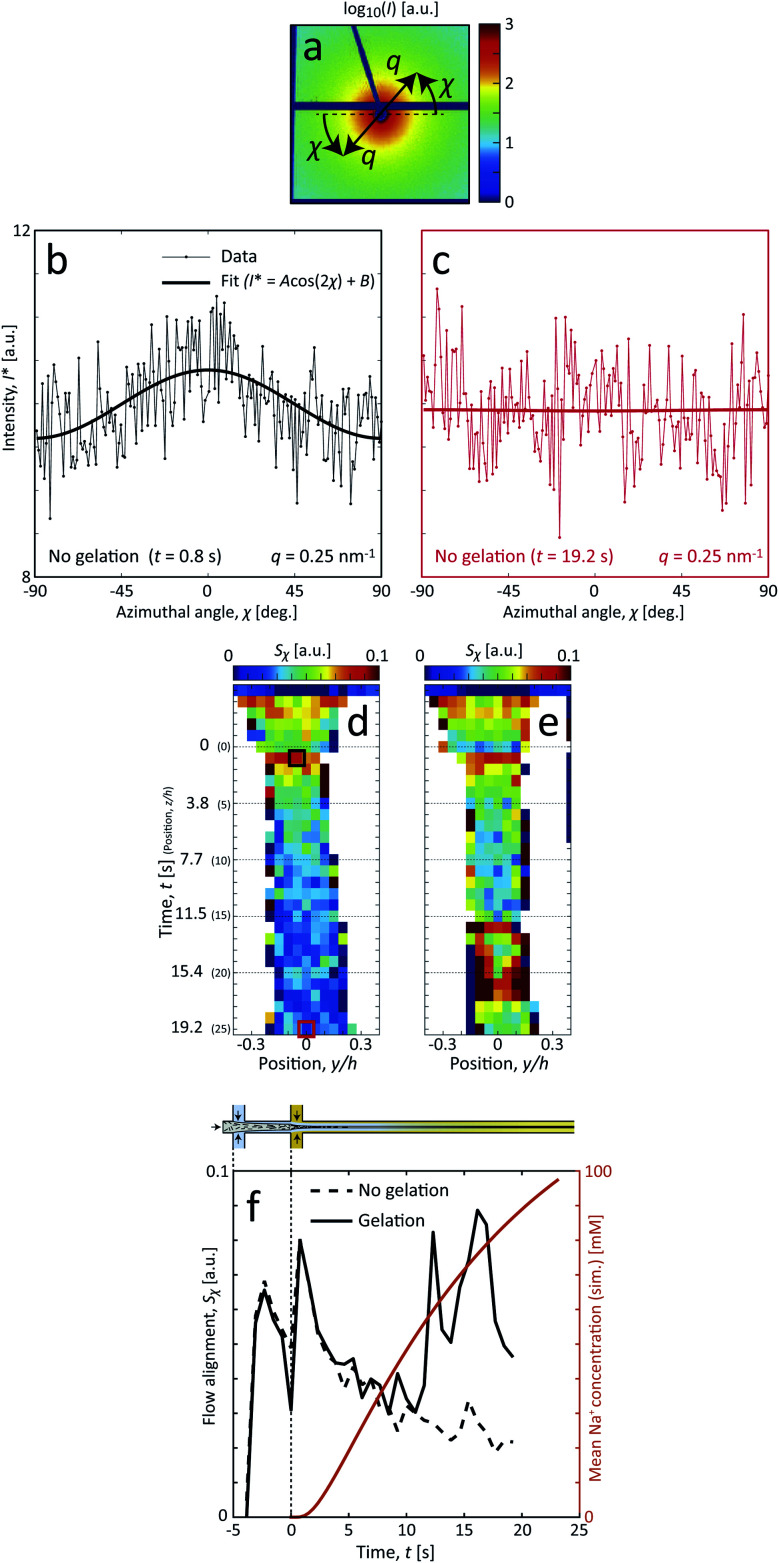
Analysis of the hydrodynamic alignment in the flow. (a) Example of a raw detector image (dark blue regions indicate masked pixels due to inter-module gaps and beamstop). (b and c) Azimuthal intensity I*(*χ*) at *q* = 0.25 nm^−1^ with no gelation at two locations in the flow; the thick line showing a fit for visual purposes. (d and e) Results of scanning-SAXS mapping the hydrodynamic alignment through the order parameter *S*_*χ*_ in the flow without and with gelation, respectively; the black and red thick squares show the positions plotted in (b and c), respectively. (f) The order parameter *S*_*χ*_ evaluated along the centerplane and compared with the simulated mean Na^+^ concentration 〈*c*〉.

The comparison between the flowing system and the pre-mixed gels highlight that the deformations inside the flow seems to have an influence on the assembly of CNFs. The big difference is that, even though the flow rates here are very low (typically 10–100 times slower than used for assembly of aligned high-performance CNF filaments^25,28^), the longer CNFs in the dispersions will be slightly aligned from the extensional flow. To study the hydrodynamic alignment in detail, the azimuthal scattering intensity I*(*χ*) is extracted as illustrated in Fig. 7a–c. After averaging and normalization in *q* ∈ [0.25, 0.45] nm^−1^ the order parameter *S*_*χ*_ is calculated the same way as done in earlier works^7,25,28,39^ with *S*_*χ*_ = 1 being a perfectly aligned system and *S*_*χ*_ = 0 for a randomly oriented system (see Methods section for details). Note here that *S*_*χ*_ < 0.1, which is much lower than in previous works with higher flow rates.

In terms of alignment, CNFs in the system with gelation initially behaves similar to the system without gelation. The two peaks at *t* = −2 s (*z*/*h* = −3) and close to *t* = 0 correspond to the positions directly after the first and second sheath flow, where extensional flow is aligning the system. After this, there is a natural decay of alignment as there are no velocity gradients in the core and Brownian rotary diffusion dominates. The effective rotary diffusion constant *D*_r_ of the aligned CNFs can be found through *S*_*χ*_ ∝ exp(−6*D*_r_*t*)^26,38^ to be *D*_r_ = 0.022 rad^2^ s^−1^ (evaluated at centerplane from *z*/*h* = 1 to 9).

The discrepancy between the two systems is obvious when the complete gel network is formed after around *t* = 6–10 s (the simulated Na^+^ concentration is 30–40 mM). When the network is formed, instead of ‘locking’ the instantaneous state at this position, as observed *e.g.* by Håkansson,^26^ the alignment rather increases slightly with downstream position. The observed larger deviations from the mean trend at *t* = 12–16 s are likely due to instabilities in the flow that are caused at the position where the network is formed. These instabilities are in turn believed to be a consequence of the combined effects due to connected network and low flow rates, causing outlet conditions to propagate upstream, including gravitational sedimentation and the need for mechanical stirring to remove gel from the outlet; both sources providing some downward force on the gel. Apart from these effects, it could also be partly caused through hydrodynamic forces from the shear flow at the interface between the CNF gel and the outer sheath flow.

**Fig. 8:**
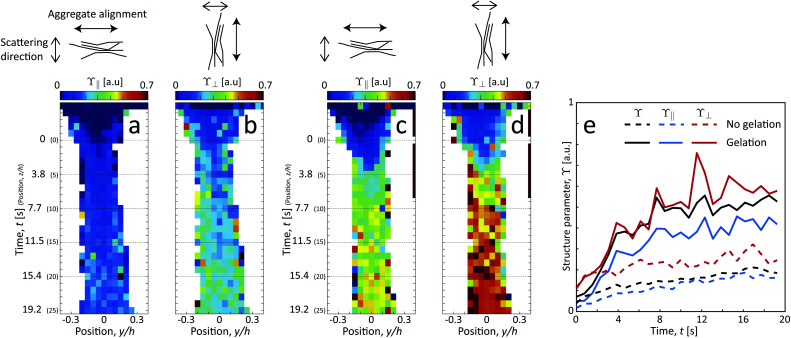
Results of scanning-SAXS mapping the orientation dependence of segmental aggregates. Structure parameter (a and c) parallel *ϒ*_∥_ and (b and d) perpendicular *ϒ*_⊥_ to the flow (a and b) without gelation and (c and d) with gelation. (e) Structure parameters *ϒ*, *ϒ*_∥_ and *ϒ*_⊥_ evaluated along the centerplane.

Since the system clearly has some degree of alignment both with and without gelation, we can also observe the orientation of the segmental aggregates by analyzing the structure parameter parallel to the flow *ϒ*_∥_ (probing structuring of segmental aggregates *perpendicular* to flow) and perpendicular to the flow *ϒ*_⊥_ (probing structuring *parallel* to flow). These are obtained by using the scattering intensities parallel *I*_∥_ = *I*(*q*, |*χ*| < 90° −Δ*χ*) and perpendicular to the flow *I*_⊥_ = *I*(*q*, |*χ*| < Δ*χ*) with Δ*χ* = 22.5°, respectively (see details in Methods section). The results are shown in Fig. 8. Both with and without gelation, the value of *ϒ*_⊥_ is constantly higher than *ϒ*_∥_, which is also in the same direction of the increased scattering from the aligned CNFs. Therefore, it can be concluded that the segmental aggregates are indeed also aligning in the flow direction.

**Fig. 9:**
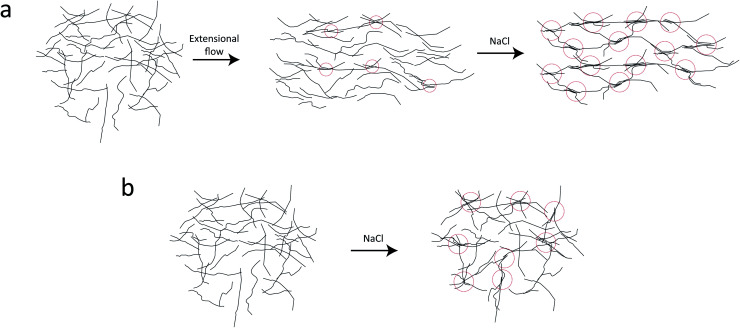
Illustration of the hypothesized scenario during CNF gel formation. (a) With extensional flow some smaller segmental aggregates (locations where nanofibrils are locally aligned and entangled; illustrated with red dashed circles) form just due to the hydrodynamic stretching of the network. This pre-stretching causes many segmental aggregates to form during gelation. (b) Gelling an isotropic system with the same concentration of salt causes the same average size of segmental aggregates but they are not as frequently occurring inside the gel.

In light of these results, we propose the following scenario of the CNF gel formation, which is illustrated in Fig. 9.

In the dispersion state, the long slender CNFs are randomly distributed in the volume and close-contact points between nanofibers are likely not with parallel nanofiber segments (Fig. 9a). The SAXS curve is therefore dominated by the form factor of individual nanofiber cross-sections. At these close-contact points, the nanofibers are still electrostatically repelled by the charged surfaces and can slide past each other allowing the CNFs to remain dispersed. When the nanofibers are subject to extensional flow, it is more likely to have close-contact points between parallel nanofiber segments, where they can become locally aggregated and entangled, forming a loose network. These regions will affect the structure factor of the scattering and can be interpreted as segmental aggregation, which is seen in the present work. This aggregation and entanglement of CNFs from extensional flow has also previously been observed to slow down Brownian dynamics.^38^ Adding salt to the stretched system, these locally aggregated/entangled regions are likely positions where nanofibers come together and form the strong segmentally crosslinked domains that build up the CNF gel. Adding more salt to the already connected network has little effect as nanofiber stiffness prevents new segmental aggregates to form.

If salt on the other hand is added to an isotropic system, the segmental aggregates are likely not consisting of locally aligned nanofiber segments and therefore not having the same effect on the structure factor (illustrated in Fig. 9b). Furthermore, it is likely that the isotropic gel needs fewer segmental aggregates to reach the point where we have a connected network dominated by the nanofiber stiffness. Both these effects are likely explanations for the slightly lower structure parameters in the pre-mixed samples at the same salt concentration. However, it should be noted that the structure factor of the pre-mixed CNF gel is also likely to be affected by the mixing conditions during gel preparation as well as the injection to the liquid sample holders. Due to the difference in the shear rate during the mixing/injection of CNFs and salt, the deformation state of the nanofibers can be affected and resulting in differences of segmental aggregation. This is a likely reason for not seeing a trend of the structure parameter with respect to the salt concentration in Fig. 6.

The comparison with our earlier work,^7^ using the same mixing experiment with cellulose nanocrystals (CNCs), highlights important fundamental differences between the two nanocellulosic systems (CNCs *versus* CNFs) during ion-induced gelation. CNCs are shorter stiffer nanoparticles, where electrostatic interactions make them move collectively in local nematic domains called *tactoids*. This aids the hydrodynamic alignment as the tactoid formation prevents Brownian rotary diffusion. The addition of salt destroys the tactoids causing Brownian rotary diffusion to dominate the system prior to gel formation, leading to an isotropic gel.^7^ For CNFs on the other hand, it has already been argued in earlier work^39^ that the collective alignment arises from mechanical interactions within the loose network rather than electrostatic interactions. It becomes even more evident in the present work, since the screened electrostatic charges of the added salt does not lead to any enhanced rotary diffusion of CNFs. Instead, it only promotes assembly of the instantaneous network, whereby an anisotropic structure arising from flow deformation can be preserved in the gel. This is the reason why remarkable properties can be achieved through the flow-focusing spinning of CNF filaments.^29^

## 3 Conclusions

In this work, we have used shear-free mixing combined with scanning-SAXS to study the time-resolved nanostructure of dispersed charge-stabilized cellulose nanofibrils (CNFs) when triggering a transition to a gel state with the addition of a salt solution. A dispersion of 0.4 wt% TEMPO-oxidized CNFs was studied, which is similar to CNF dispersions used to produce strong stiff cellulose filaments by a process almost identical to this experiment.^25,26,28^

The nanostructure was characterized by extracting the structure factor and alignment from the SAXS results. The structure factor of CNFs can be attributed to the size and amount of segmental aggregates in the nanofibrillar network.^6^ These are described as regions of locally aligned and/or entangled nanofibril segments. The extensional flow in the flow-focusing device promotes a situation of more segmental aggregation, although the nanofibrils will cause only a loosely entangled network due to electrostatic repulsion without the ionic gelation agent. However, by adding salt solution to the stretched network, higher segmental aggregation seems to take place than compared to the pre-mixed (isotropic) CNF gels at the same salt concentration. It is also found that the salt concentration has an effect of the size of segmental aggregates, but only up to the point where the complete network is formed, which is where the Na^+^ concentration is roughly twice the surface charge of the CNFs. The indication that the gel structure is mainly modified through the deformation state prior to gelation would support ideas presented earlier^29^ of why wet-spinning based on shear-free flow-focusing can create stronger CNF networks. However, to conclude exactly the relationship between deformation states and segmental aggregation, more experiments would be needed.

The present work in combination with our earlier work^7^ highlight the importance of selecting the right processing strategy when fabricating materials from nanocellulose. For processes involving ion-induced assembly of CNCs, it was clear that the time scale of assembly must be shorter than the time scale of *enhanced* rotary diffusion arising from tactoid breakup, which could be tricky to achieve practically. For processes involving CNFs, on the other hand, rotary diffusion is mainly determined by thermal motion and mechanical interactions within the loose network. Therefore, the main strategy is to reduce the *inherent* rotary diffusion of the dispersion to maximize the alignment and segmental aggregation prior to the ion-induced assembly.

## 4 Materials and methods

### 4.1 Sample preparation

Aqueous slurries of cellulose nanofibrils (CNFs) from bleached wood pulp were purchased from the Process Development Center of University of Maine. The preparation of the electrostatically stabilized dispersions in this study was identical to our previously described procedure^39^ and no further characterization was performed in this work. The CNF dispersion was prepared from a slurry with 1.1 wt% TEMPO-oxidized nanofibrils in water. The charge was 1.5 mmol carboxylate (COO^−^) per gram of solid material. The slurry was diluted with de-ionized (DI) water to a concentration of approximately 0.4 wt%, then mixed and passed through a high pressure homogenizer five times at a pressure of 200 bar. The dispersion was then filtered (40 μm filter) and ultrasonicated overnight (>12 h), after which it was assumed to be free of larger aggregates. The final concentration was subsequently determined through gravimetric analysis, revealing that the change in concentration from the initial dilution was negligible. At the final concentration of 0.4 wt%, the charge concentration was 6 mM COO^−^. The *ζ*-potential was measured (NanoBrook Omni, Brookhaven Instrument Inc.) to be − 45 ± 8.5 mV. Through transmission electron microscopy (TEM) of the material, the lengths of CNFs were previously determined^39^ to be between 200–1000 nm. Using a parallelepiped model to fit the isotropic SAXS data, it was previously determined^39^ that the mean cross sectional dimensions of the CNFs were 5.6 × 2.0 nm^2^.

### 4.2 Flow cell and fluid distribution

The flow cell in this study is identical to the one used in our earlier work.^7^ The flow-focusing geometry, illustrated in Fig. 1, was created by milling 1 mm wide channels in a 1 mm thick aluminum plate, creating the quadratic cross-section with side *h* = 1 mm. The channel plate was sandwiched between 150 μm thick COC films (Tekni-plex 8007 X-04) and was mounted together with outer aluminum plates (see Fig. S1 in ESI†). The distance between the two sheath flow channels was 5 mm. The main flow direction was in the same direction as gravity and the flow eventually went straight into an outlet container with DI-water. The flow was driven by three syringe pumps (NE-4000) with 1 mL syringes, each at a flow rate of *Q* = 0.5 mL h^−1^. The flow setup is illustrated in Fig. S2 in ESI† along with further details of the experimental setup.

### 4.3 *In situ* SAXS experiments

The *in situ* SAXS experiments were performed at the LiX beamline (16-ID) in the NSLS-II, Brookhaven National Laboratory, USA, and are identical to our previously reported experiments.^7^ The flow cell was mounted on a translation stage in the path of the beam to allow for scanning in both *y*- and *z*-directions (see Fig. S3 in ESI† for photos of the experimental setup). The flow was running continuously while taking one image with 1 s exposure at various positions in both *y*- and *z*-directions. Each scan of 17 horizontal points at 31 different downstream locations (527 points in total) took approximately 30 min. In these measurements, the core interface was monitored with an on-axis camera during the entire measurement to ensure the flow stability throughout the measurement. For the pre-mixed gels, the samples were injected into liquid sample holders, with window materials made from mica, which were scanned at five different positions with 1 s exposure at each position. A second image was scanned directly afterwards at the same position to ensure that there was no beam damage to the sample. The wavelength was *λ* = 0.9 Å and the sample-detector-distance was 3.6 m. The beam size was approximately 50 × 50 μm^2^. The scattered X-rays were recorded on a Pilatus 1 M detector with pixel size 172 × 172 μm^2^ covering a range of *q* = 0.06–2 nm^−1^ with scattering vector magnitude *q* = (4π/*λ*)sin *θ* (the angle between incident and scattered light is 2*θ*). The background scattering intensity was obtained by flowing only DI water in the channel and subtracted from the experimental data. Prior to this subtraction, the background intensity was scaled with a factor (close to one) corresponding to the ratio of the recorded transmitted beam. The SAXS intensity *I*(*q*_*y*_, *q*_*z*_) was converted into polar coordinates *I*(*q*, *χ*) with azimuthal angle *χ* in the detector plane (*χ* = 0 in the *y*-direction). The pixels covered by inter-module gaps and the beamstop were corrected using the fact that *I*(*q*_*y*_, *q*_*z*_) = *I*(−*q*_*y*_, −*q*_*z*_). The 1D SAXS data *I*(*q*) was extracted by averaging the 2D data over all angles *χ*.

### 4.4 Modelling SAXS of segmental aggregates

The simulations of the segmental aggregates assumes parallel segments of fibrils with a square cross-section with side *A*_CNF_ = 2.5 nm similar to the true cross section of an elementary cellulose microfibril.^9^ However, the actual cross-section (form factor) is not important as the model is used to capture the structure factor. The length of the fibrils provide negligible contribution to the scattering in the experimental *q*-range and are assumed to have infinite length. The scattering intensity is thus only determined by the cross-sectional distribution of fibrils within the aggregate. Firstly the simulated detector coordinates are defined as *q*_*y*_, *q*_*z*_ ∈ [−0.2, 0.2] nm^−1^ with 500 pixels in each direction. The positions ***r***_*i*_ = (*y*, *z*) of *N* fibrils are sampled randomly within a circular region of radius *R* representing the size of the segmental aggregate (see Fig. 4). The 2D structure factor is calculated through:1
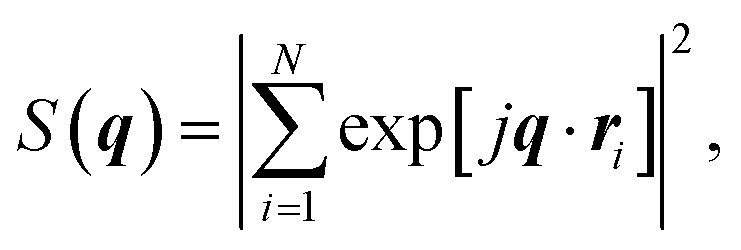
with 
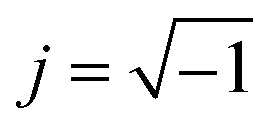
. The simulated intensity from the cross-section *I*_cs_(***q***) = *P*(***q***)*S*(***q***) is found by multiplication of the form factor for a square (parallelepiped aligned out of plane^44,45^):2
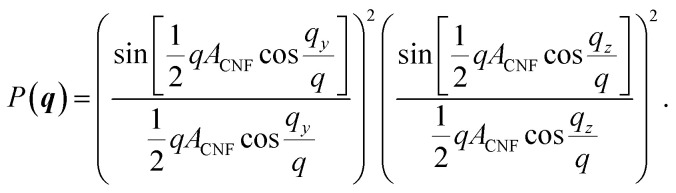


The isotropic intensity curve of a segmental aggregate is found by averaging the intensities over spherical shells in the reciprocal domain:3
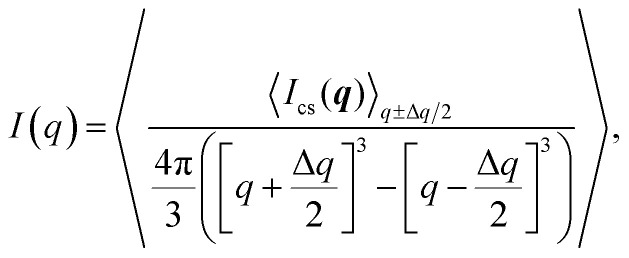
where the inner brackets indicate the mean intensity of all pixels within a circular section of *q* ±Δ*q*/2 (Δ*q* is chosen as the same size as the pixel size on the simulated detector) and the outer brackets denote an average over 100 different configurations for a given *R* and *N*. Normalized intensity curves are obtained by I* = *I*/*N* and the isotropic normalized structure factor is obtained through S* = I*/P*, where the normalized isotropic form factor P* is obtained through the same procedure as above using *S*(***q***) = 1. The effect of volume fraction of aggregates *Φ*_SA_ is studied by using 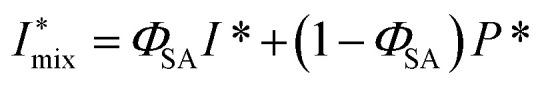 and analyzing the structure factor 
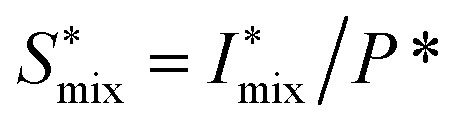
 as before.

### 4.5 Post-processing of isotropic SAXS data

The experimental form factor *P*(*q*) of dilute CNFs was determined from the isotropic (low flow rate) data in earlier work^39^ using the same CNF suspension at 0.4 wt%. The intensity data *I*(*q*) from five different measurements was fitted with a spline interpolation in Matlab R2018b to create the curves of *P*(*q*) used to evaluate the structure of the present systems (see Fig. 2b). The isotropic intensity data *I*(*q*) is used to estimate the invariant Q*, which is a measure of the total scattering power of the system and is typically proportional to the amount of particles in the beam and calculated through:4
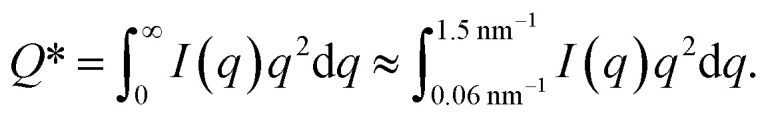


The normalized intensity curves (normalization denoted with asterisk) from the experiment is obtained through I* = *I*/Q*. The structure parameter S* is obtained through S* = I*/P* and is fitted in the range *q* ∈ [0.06, 0.4] nm^−1^ to a function of form:

5 S* = *Y* exp(−*ξ*^2^*q*^2^) + *B*,

to obtain the structure factor *ϒ* and correlation length *ξ*. Baseline *B* is also fitted although the parameter naturally is very close to 1. The radius of the segmental aggregates is obtained through a relationship *ξ* = 0.4*R* demonstrated in Fig. 4. The centerplane positions *y*_cp_ are determined by the maximum of Q* at each downstream position *z*, and the centerplane parameters are obtained through a mean of positions *y*_cp_ ± 0.05 mm.

### 4.6 Post-processing of anisotropic SAXS data

The anisotropic post-processing of SAXS data followed the same procedure as described previously.^7^ To obtain the orientation distribution function *Ψ*_*χ*_ of the CNFs from the 2D SAXS data, the same method was applied as described earlier.^39^ In brief, the isotropic contribution to the scattering *I*_iso_(*q*) was subtracted from the 2D data *I*(*q*, *χ*), with subsequent normalization at each *q* and averaging over a given *q*-range resulting in *Ψ*_*χ*_. The isotropic data *I*_iso_(*q*) was determined by *I*_iso_(*q*) = *I*(*q*_*y*_ = 0, *q*_*z*_ = *q*) from a highly aligned system achieved through single flow focusing at high flow rates. Since the same dispersions were used here, *I*_iso_(*q*) was the same as in earlier work.^39^ Prior to subtraction, the isotropic contribution was scaled using the invariant Q*. Once *Ψ*_*χ*_ was extracted, the alignment was characterized with the order parameter *S*_*χ*_, defined as:6
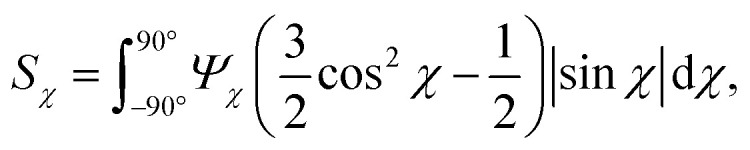
where *Ψ*_*χ*_ is normalized according to:7
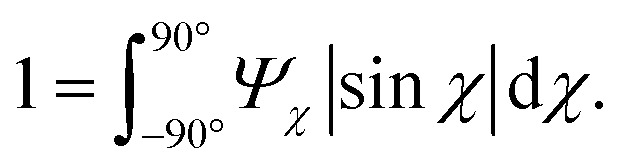


The order parameter *S*_*χ*_ is obtained in a region of *q* ∈ [0.25, 0.45] nm^−1^, where scattering is dominated by the cross-section of individual CNFs.

To study the orientation dependence of the structure parameter, firstly the intensity curves parallel and perpendicular directions were extracted as *I*_∥_(*q*) = 〈*I*(*q*,*χ*)〉_|*χ*|<90°−Δ*χ*_ and *I*_⊥_(*q*) = 〈*I*(*q*,*χ*)〉_|*χ*|<Δ*χ*_ with Δ*χ* = 22.5°, respectively, where the brackets denote averaging over angles in the subscript. The parallel and perpendicular structure factors are obtained through normalization and dividing with the isotropic form factor, *i.e.*
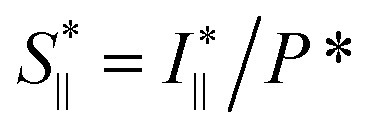
 and 
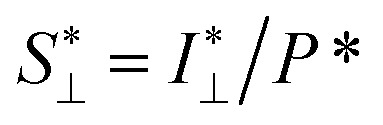
. The structure factors are then fitted in the same manner as before to obtain structure parameters *ϒ*_⊥_ and *ϒ*_∥_. Note that the normalization procedure effectively removes the influence of the alignment of the CNF cross-sections (characterized with *S*_*χ*_).

### 4.7 Ion diffusion simulations

The ion diffusion simulations were identical to our previously reported procedure.^7^ The channel cross-section was assumed to be cylindrical with outer radius 
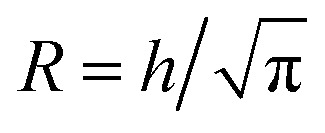
, where the radius of the core flow is given by *R*_c_ and the boundary between buffer and salt solution was given by radius *R*_2_. Both core velocity *V* = 1.3 mm s^−1^ and core flow radius *R*_c_ = 0.185 mm were determined by complementary experiments using POM (see Fig. S4 in ESI†). The buffer radius *R*_2_ = 0.32 mm is identical to the one estimated in earlier work.^7^ To simulate the ion concentration profile in the channel, the diffusion equation for ion concentration *c* at radial coordinate *r* (from centerline) was simulated in time according to:8
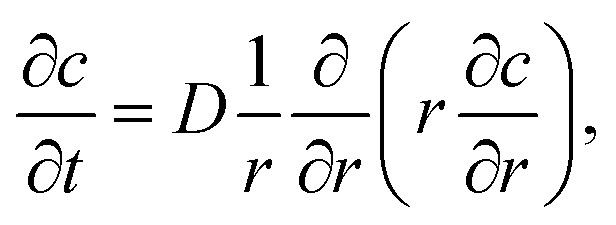
where *D*_ion_ = 1.3 × 10^−9^ m^2^ s^−1^ is the diffusivity Na^+^ in water. Initial conditions are set to *c*(0 ≤ *r* < *R*_2_, *t* = 0) = 0, *c*(*R*_2_ ≤ *r* ≤ *R*, *t* = 0) = *c*_0_ and ion concentration *c*_0_ = 200 mM in outer sheath fluid. The concentration gradient is further set to zero at the boundaries. The mean concentration in the core is calculated through:9
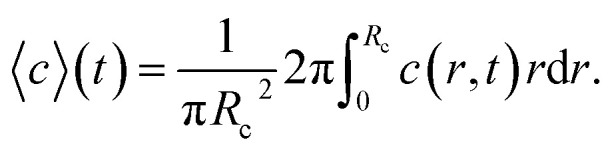


Time instances *t* are then easily converted to spatial coordinates *z* through *z* = *Vt*. The results from the ion diffusion simulations are illustrated in Fig. S5 in ESI.†

## Conflicts of interest

There are no conflicts to declare.

## Supplementary Material

NA-003-D1NA00236H-s001

NA-003-D1NA00236H-s002
