# Supplementary material for: Understanding ion-induced assembly of cellulose nanofibrillar gels through shear-free mixing and in situ scanning-SAXS
Source: Nanoscale Adv. 2021 Jul 19;3(17):4940–51. doi: 10.1039/d1na00236h (PMC8386416; doi:10.1039/d1na00236h)
Supplement: NA-003-D1NA00236H-s002 [file NA-003-D1NA00236H-s002.pdf]

Electronic Supplementary Material (ESI) for Nanoscale Advances.
This journal is © The Royal Society of Chemistry 2021

Understanding ion-induced assembly of cellulose nanofibrillar gels through shear-free mixing and *in situ* scanning-SAXS

Supporting Information

Tomas Rosén,[*,†,‡,¶] Ruifu Wang,[†] HongRui He,[†] Chengbo Zhan,[†] Shirish Chodankar,[§] and Benjamin S. Hsiao[*,†]

†*Department of Chemistry, Stony Brook University, Stony Brook, New York 11794-3400, United States*

‡*Department of Fiber and Polymer Technology, KTH Royal Institute of Technology, SE-100 44 Stockholm, Sweden*

¶*Wallenberg Wood Science Center, KTH Royal Institute of Technology, SE-100 44 Stockholm, Sweden*

§*National Synchrotron Light Source II, Brookhaven National Lab, Upton, NY, USA*

E-mail: trosen@kth.se; benjamin.hsiao@stonybrook.edu

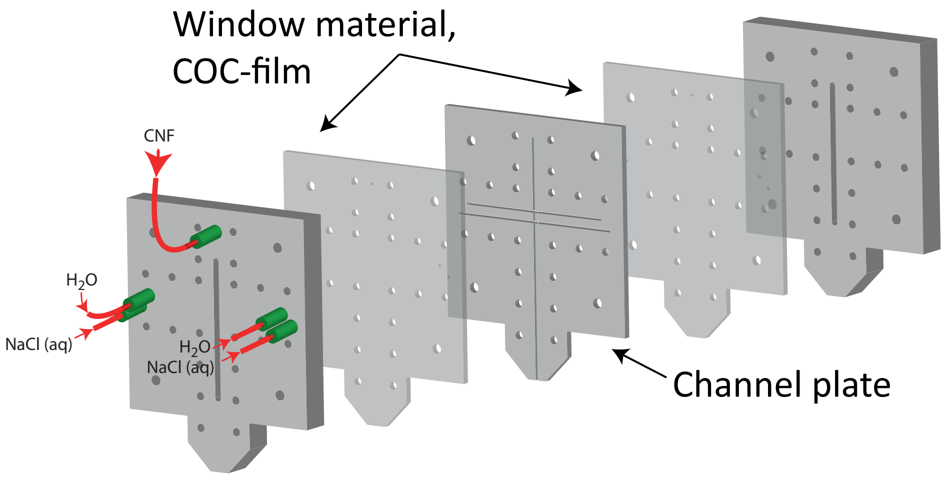

Figure 1: Illustration of the flow cell, which is identical to the one used by Rosén et al.[1]. It consists of an aluminum channel plate sandwiched between two transparent COC-films that are all mounted together with two outer thicker aluminum plates. Reproduced from Rosén et al.[1] with permission from the Royal Society of Chemistry.

Flow cell

Fig 1 shows the flow cell used for the experiments, which is identical to the one used by Rosén et al.[1]. The double-focusing channel is milled out of a 1 mm thick aluminum plate, which is sandiwiched between two COC-films that both provide optical access to the mixing region and act as walls to the flow. The sandwich is mounted with two outer 10 mm thick aluminum plates where connections for the fluid are attached at the front plate facing the X-ray beam.

Flow setup

Fig 2 shows the setup of the flow equipment, which is identical to Rosén et al.[1]. Three syringe pumps (NE-4000) are driving the main flow (with flow rates Q_1, Q_2 and Q_3) with 1 mL syringes. To fill up tubing prior to the experiment and push out bubbles, two additional syringe pumps with larger 20 mL syringes are connected to the core and 2nd sheath flow, respectively. To avoid gelation in the channel while not measuring as well as flushing the channels from any gel accumulation, two additional pumps are connected to the 1st sheath

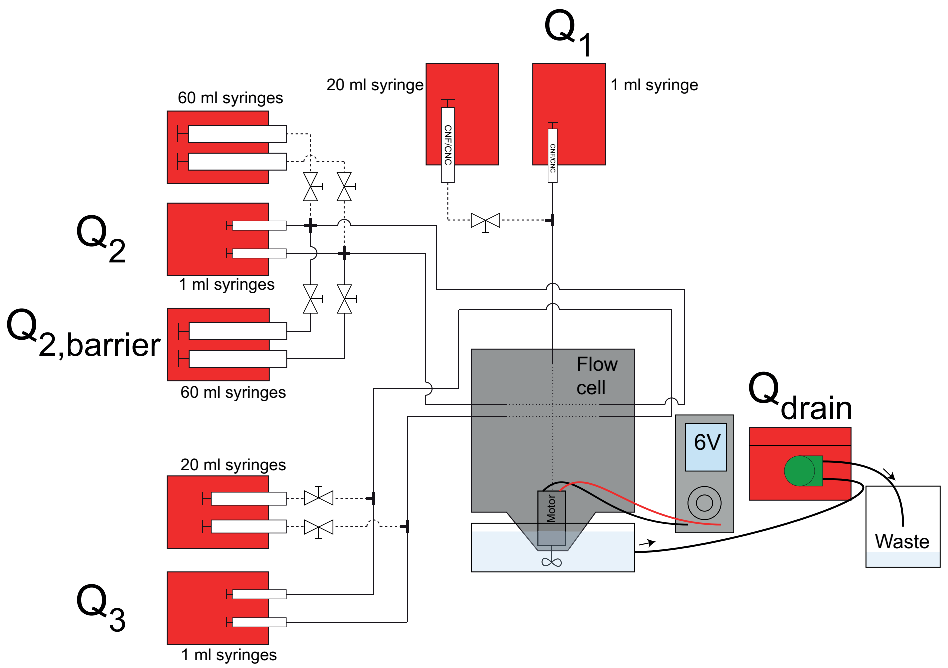

Figure 2: Illustration of the fluid distribution to the experiments, which is identical to Rosén et al.[1]. To be able to run at low flow rates, small 1 mL syringes are driving the main flow. However, larger syringes are also connected to the system to be able to fill up the tubing as well as flush the flow cell with higher flow rates. To maintain a constant level in the outlet container, a peristaltic pump is continously removing water from the container. A small motor is mounted at the outlet to remove excess gel that otherwise could result in blockage. The pumps flow rates $Q_1, Q_2, Q_{2,\mathrm{barrier}}, Q_3$ and Q_{drain} are controlled remotely from outside the experimental hutch. Reproduced from Rosén et al.[1] with permission from the Royal Society of Chemistry.

flow, each with two large 60 mL syringes with water. To increase the stiffness of the system and avoid potential bubbles in the large syringes to store energy and act as springs in the fluidic system, manual shut-off valves are placed according to the figure. During experiment all valves were shut to the syringe pumps, except for the valves to one of the 1st sheath flow pumps (with flow rate denoted as $Q_{2,\mathrm{barrier}}$), in order to quickly flush the system.

A peristaltic pump (with flow rate denoted as Q_{drain}) is used to drain the outlet container and keeping the level stationary and a small 6V motor rotating at around 5 rpm is slowly removing gel at the outlet without disturbing the flow upstream.

During the SAXS experiments, the three syringe pumps with the 1 mL syringes (flow rates Q_1, Q_2 and Q_3) as well as the barrier-pump for the 1st sheath flow and the peristaltic

pump are controlled remotely from outside the experimental hutch. In this experiment, all the pumps with the 1 mL syringes are running at the same flow rate $Q_1 = Q_2 = Q_3 = Q$, meaning that the perstaltic pump is set to $Q_{\text{drain}} = 5Q + 2Q_{2,\text{barrier}}$ to keep the level constant.

The normal sequence of flow rates during the scan is as following:

1. The 1st sheath is set to $Q_{2,\text{barrier}} = 50$ mL/h, to ensure no gelation in the channel.
2. All primary flows are set to the desired flow rates $Q_1 = Q_2 = Q_3 = Q$, where the experiments reported in the main manuscript is running at $Q = 0.5$ mL/h.
3. $Q_{2,\text{barrier}}$ is set to zero and the flow is allowed to stabilize for a couple of minutes prior to measurement.
4. During the scanning-SAXS measurement, the system is running continously, while data is collected from the different y- and z-locations in the flow.
5. To remove possible gel that might have accumulated during the scan, the channel is flushed with $Q_{2,\text{barrier}} = 5000$ mL/h for 1 s directly after the measurement and then set to $Q_{2,\text{barrier}} = 50$ mL/h again.

Experimental setup for SAXS experiments

Figure 3 shows the experimental setup of the *in situ* scanning-SAXS experiments at the LiX beamline, NSLS-II, Brookhaven National Laboratory, USA. Note that the setup is identical to the setup used by Rosén et al.[1].

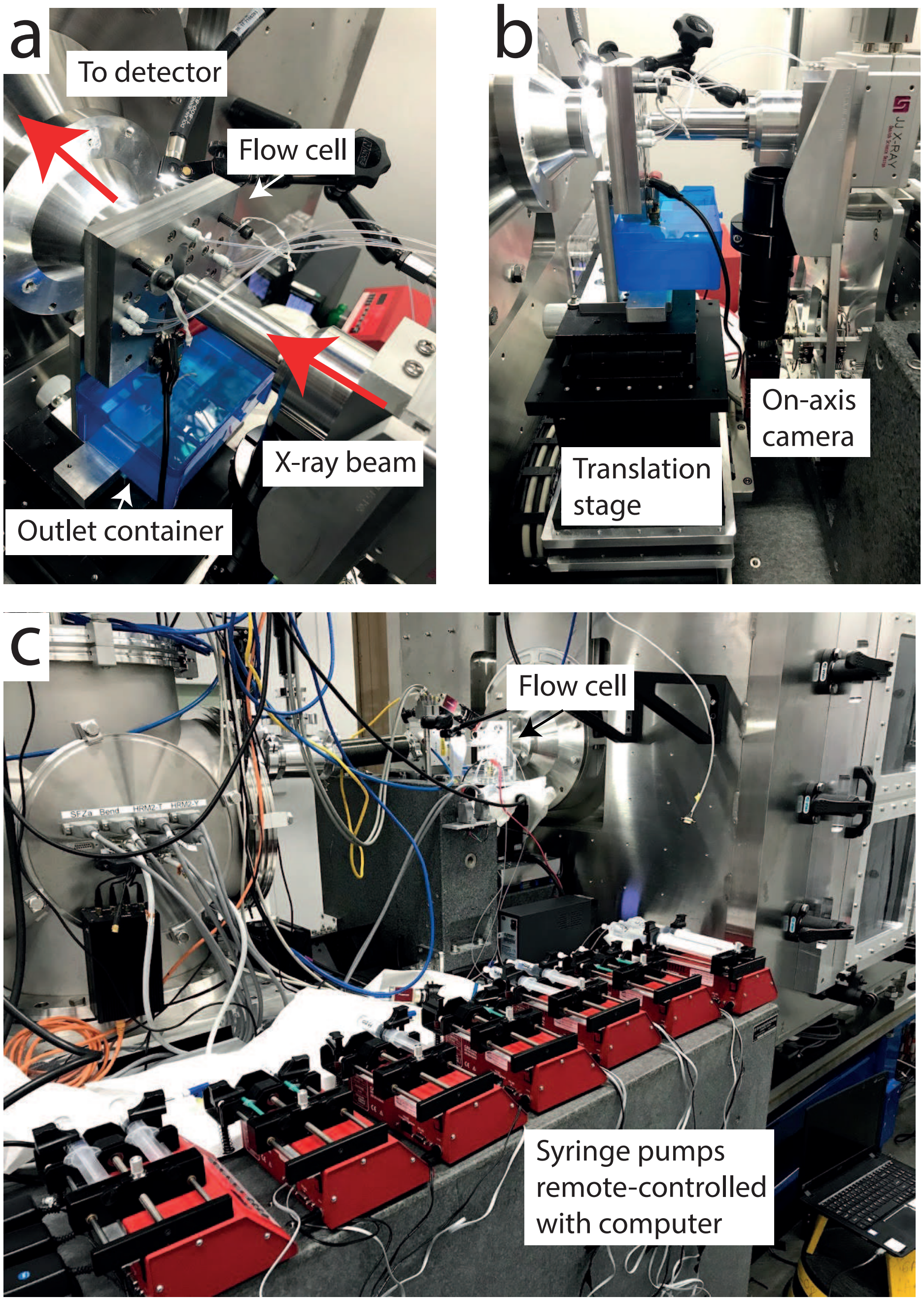

Figure 3: Photos from the experimental setup at the LiX beamline (16-ID), NSLS-II, Brookhaven National Laboratory, USA. The experimental setup is identical to Rosén et al.[1]. (a)-(b) shows photos from different directions of the flow cell; (c) photo of the pump setup at the beamline. Reproduced from Rosén et al.[1] with permission from the Royal Society of Chemistry.

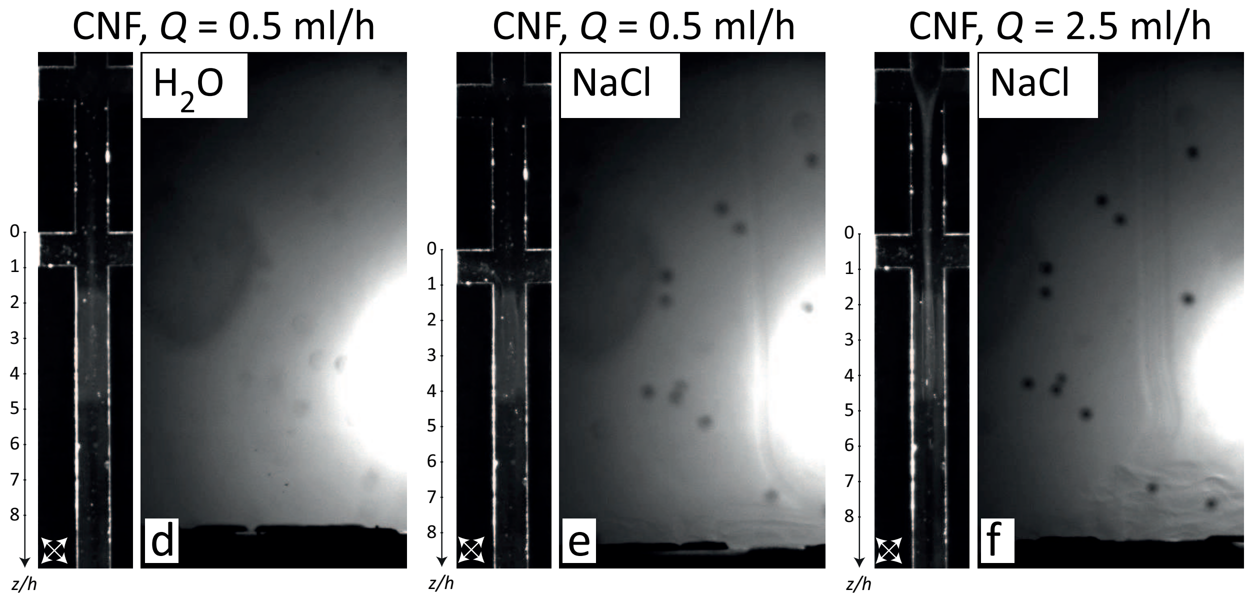

Figure 4: Images from the *in situ* polarized optical microscopy (POM) experiments (corresponding videos provided as a suuplementary file). The left side shows the POM intensity inside the channel while the right side shows the CNF dispersion exiting the outlet. (a) Q = 1 mL/h with no gelation (water in the 2nd sheath flow). (b) Q = 1 mL/h with the NaCl solution as gelation agent. (c) Q = 2.5 mL/h with the NaCl solution as gelation agent.

Supporting POM experiments

Just as in our earlier work,[1] the flow was studied with polarized optical microscopy (POM) in order to ensure that there is a gel exiting the channel at the given flow conditions as well as to determine the plug velocity profile with core flow radius R_1 and velocity V. In Fig. 4 images from the POM experiment are shown with the left side showing the flow-induced birefringence (due to hydrodynamically aligned CNFs) in the channel and the right side showing the flow as it exists the flow cell into an outlet container with water. A video illustrating these experiments is provided as a supplementary file.

Given the low flow rates and low concentration of the CNF dispersion, the birefringence is not really visible at the experimental flow rate of Q = 0.5 mL/h and there is no visible difference in the channel in the case without gelation (Fig. 4a) and with gelation (Fig. 4b). Without gelation, we cannot observe the dispersion as it exists the channel, probably as the concentration gradient of nanofibers is more smeared out as nanofibers diffusing into the

water. With the CNF gelation at the experimental flow rate $Q = 0.5$ mL/h (Fig. 4b), the gel thread is observed and accumulating on the bottom of the container. At a 5X higher flow rate $Q = 2.5$ mL/h (Fig. 4c), some alignment is seen by the birefringence in the channel. However, it seems that the gel is rather form outside the flow cell formed rather than inside the flow cell, similar to the dispersion with CNCs as observed by Rosén et al. [1].

Just like with the CNCs,[1] we could also observe small impurities in the CNF dispersions. By tracking these, it was straightforward to confirm that the flow is a plug flow where all impurities moving with a constant velocity. This allowed us also to experimentally determine the core velocity V and the projected radius $R_{1,y}$. Since we know the core flow rate Q_1, the core radius in the viewing direction $R_{1,x}$ can be calculated through $Q_1 = \pi R_{1,x} R_{1,y} V$. An illustration of the procedure is included in the supplementary video.

Estimating the ion concentration distribution in flow-focusing mixing

Simulations of the diffusion equation

Using the experimental values of V and R_1 (mean of $R_{1,x}$ and $R_{1,y}$), the ion concentration was estimated (with buffer radius $R_2/h = 0.32$ estimated by Rosén et al. [1]) using the procedure in the main manuscript. The Na^+ concentration is estimated at different radial positions and downstream locations in Fig. 5a-b. From the simulated concentration profiles, three values were determined at different downstream positions: the centerline concentration (c_{CL}), the interface concentration at $r = R_1$ (c_{IF}) and the mean concentration in the core ($\langle c \rangle$), where the latter is used for comparison in the scanning-SAXS experiments (Figs. 4 and 5 in the main manuscript).

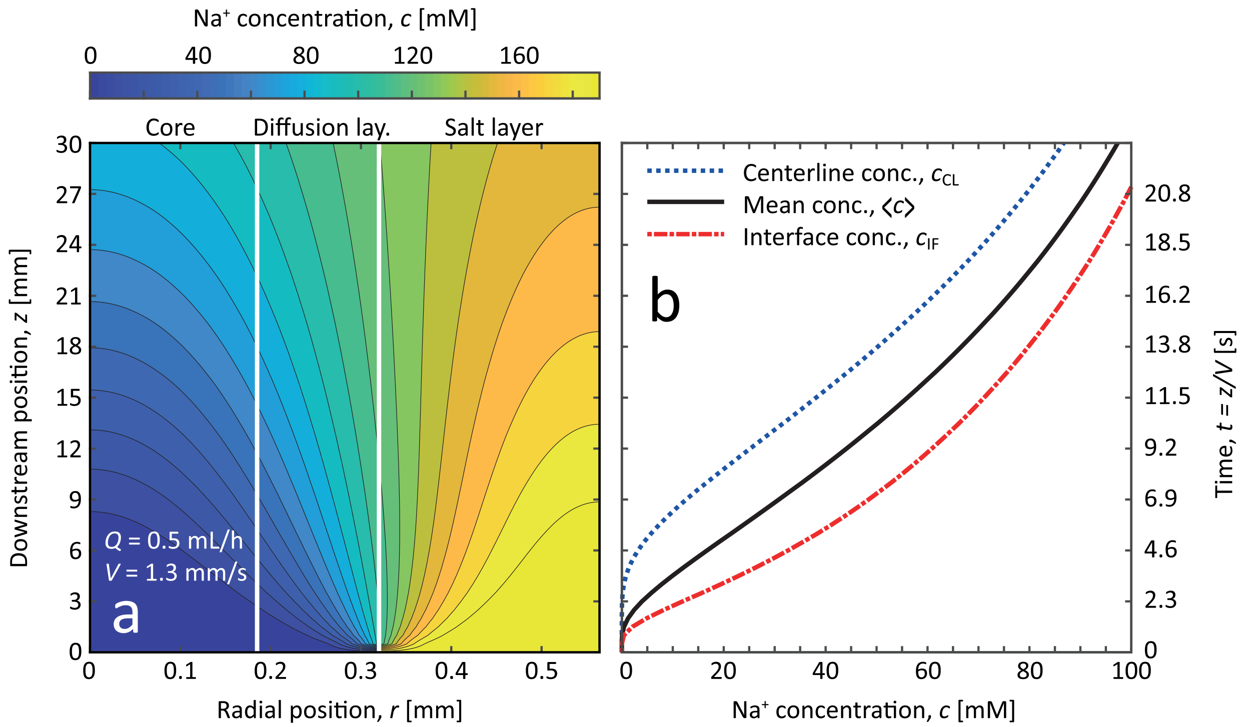

Figure 5: Concentration of Na^+ at different downstream locations z and radial positions r according to the procedure described in the work by Rosén et al.[1] using the experimental values of core velocity V and radius R_1. (a) Concentration profile and (b) centerline, mean and interface concentrations at $Q = 1.3$ mL/h. Contours in (a) separated with 10 mM.

References

(1) Rosén, T.; Wang, R.; He, H.; Zhan, C.; Chodankar, S.; Hsiao, B. S. Shear-free mixing to achieve accurate temporospatial nanoscale kinetics through scanning-SAXS: Ion-induced phase transition of dispersed cellulose nanocrystals. *Lab Chip* **2021**, *21*, 1084–1095.
